# A substantial increase of the impact factor

**DOI:** 10.3109/03009734.2012.725511

**Published:** 2012-10-30

**Authors:** Arne Andersson, Gunnar Ronquist

**Affiliations:** ^1^Editor, basic sciences; ^2^Editor, clinical sciences

When we contracted, some 4 years ago, an established publisher (Informa Healthcare) for the publication of our journal, we had to set a reasonable goal for our achievements. Perhaps, not surprisingly, we looked at the so much debated impact factor institute and found that by reaching the critical 1.0 level—used by our local faculty in their budgetary system—a realistic and meaningful aim had been set. As a physical token of this planning we promised our editorial board members a champagne party the day we had brought about this considerable effort. So, it is our great pleasure to announce that we have succeeded. As published late June by Thomson Reuters (TR) in their annual report, our 2-year impact factor has risen to 1.063, a more than 70% increase as compared with last year’s figure (Table I). The increase of the 5-year impact factor figure was less impressive, indicating that the citations of the last 2 years’ articles have contributed to a higher extent. We can also see a sharp increase of the immediacy index, telling us that many of our articles are cited very soon after they have been released on the ‘Early Online’ site at the website of the journal. It is also interesting to see that the numbers of total cites in both databases (T&R and SCOPUS) have increased substantially fulfilling our objective to be cited once every day all days of the year. As we pointed out in our latest editorial on the performance of *UJMS* (1), we have managed to keep up the percentage of our papers that indeed become cited—75%–80% after 3–5 years—which is very close to figures for other more well-known and well-reputed scientific journals. Concerning the rank figures, it can be seen that we are close to entering the best half of all journals belonging to the category ‘Medicine, general and internal’. A reasonable next goal of our journal will be to establish ourselves in the better half.

**Table I.** 5-year trends in the performance of the journal.

Year	Impact factor 2-year TR	Impact factor 5-year TR	Immediacy index TR	Rank TR	Total cites TR	Total cites SCOPUS	% not-cited articles SCOPUS
2007	0.500	0.570	0.033	79/100	229	247	23
2008	0.677	0.683	0.067	82/107	262	274	20
2009	0.733	0.745	0.083	90/103	259	281	26
2010	0.636	0.623	0.209	99/153	271	310	45
2011	1.063	0.770	0.395	80/153	347	378	63

Of course, these increases in different parameters all depend on the fact that we have successfully selected good papers for publication. In that process our editorial board members and reviewers have all done a tremendous job. We take this opportunity to thank them all! The champagne expert has been called upon. In order to make this selection successful a good submission rate is a prerequisite. We have had some 150–200 original papers submitted each year, and we accept about 50 manuscripts for publication. So almost three out of four papers have to be rejected, and that is a cumbersome process.

In this context it is interesting to see how one single article can be the difference between failure and success. Amongst the citations for the 2011 impact factor figure there is one paper that stands out. The review by Niklas Nordquist and Lars Oreland on ‘Serotonin, genetic variability, behaviour, and psychiatric disorders’ (2) was cited 16 times last year and then was responsible for more than 20% of the citations to the 2010 and 2011 volumes last year. At present (August 2012) that particular article has been cited 24 times. An article cited that much belongs to the 1% most-cited publications (3). For that type of calculations citations during the publication year and the two following years are counted. The number of cites for the 1% limit is 22, and for the corresponding 10% limit 8 citations. We can foresee that a couple of our articles will reach that lower level as well since almost half a year of citation collection still remains.

Apart from the fact that the quality of our published papers undoubtedly has increased, there might be at least one more issue that speaks in favour of our journal when authors look for an attractive publication source. Also the ‘open access’ entrance on the publication arena has become more and more important. Ever since the start of the electronic publication of our journal, all papers—old (since a few years back) as well as brand-new—have been freely accessible at our website. This in combination with the fact that we have not charged our authors any type of publication fees has made our journal quite unique. Most probably, however, we will have to change this somewhat in the sense that we will introduce some sort of handling fee for our published articles. At least for non-faculty members. This then should be interpreted as one more invitation to our researchers at the faculty to submit their reports to our journal. Last year some 40% of our published papers came from Uppsala-based teams. We would very much welcome more of them.

## References

[1] Editorial (2010). Lessons from the recent Journal Citation Report figures. Ups J Med Sci.

[2] Nordquist N, Oreland L (2010). Serotonin, genetic variability, behaviour, and psychiatric disorders—a review. Ups J Med Sci.

[3] Karlsson S (2010). Den svenska produktionen av högt citerade vetenskapliga publikationer. Vetenskapsrådets Lilla Rapportserie.

